# Ultra-Wide-Field Fluorescein Angiography in Microscopic Polyangiitis

**DOI:** 10.1155/2016/9834684

**Published:** 2016-10-30

**Authors:** Philip P. Storey, Shannon A. Philander, Anna Ter-Zakarian, Narsing A. Rao, Damien C. Rodger

**Affiliations:** USC Roski Eye Institute, University of Southern California, Los Angeles, CA, USA

## Abstract

A 25-year-old Hispanic female presented with 5 months of dry eyes and 2 months of bilateral photophobia and decreased vision. On examination, she had bilateral anterior uveitis and mild disc edema of the left eye. A complete infectious and inflammatory work-up was positive for elevated antinuclear antibodies and p-ANCA, leading to a diagnosis of microscopic polyangiitis. One year after initial treatment and steroid taper, an ultra-wide-field fluorescein angiography revealed peripheral vasculitis, outside of the standard traditional field of view, leading to an increase in immunomodulatory therapy and illustrating the utility of wide-field angiography for managing patients with uveitis.

## 1. Introduction

Fluorescein angiography (FA) has become an essential tool for evaluating and managing noninfectious retinal vasculitis. Based on the evaluation of a patient's retinal imaging, clinical evaluation, and symptoms, treatments are titrated to control inflammation while keeping side effects minimized. Fluorescein angiography allows for direct visualization of retinal vasculitis through evaluation of vascular leakage and capillary dropout. However, traditional FA is limited by the inability to image more than 50 degrees per image as well as decreased image quality of peripheral retina caused by optical aberrations. Multiple images can be overlapped and a montage created but the traditional 7 standard image field only extends up to 75 degrees, leaving large swaths of peripheral retina unphotographed [1].

Ultra-wide-field fluorescein angiography allows for capturing up to 200 degrees of the fundus in a single image. Wide-field imaging also enables simultaneous angiographic visualization of most of the retina, which the traditional FA montage does not allow. A limited number of previous reports have evaluated ultra-wide-field fluorescein angiography in noninfectious retinal vasculitis [2–6]. In this case report, we present wide-field imaging of a patient with microscopic polyangiitis, which proved to be clinically useful in our patient's management.

## 2. Case Report

A 25-year-old Hispanic female initially presented to Los Angeles County Hospital with 5 months of dry eyes and 2 months of bilateral photophobia, eye redness, and gradually decreasing vision worse in her right eye than her left. Her past medical history was significant for childhood asthma, which had resolved. Current medications included loratadine by mouth, restasis twice a day in each eye, and artificial tears as needed. On presentation, her best-corrected Snellen visual acuity was 20/400 in the right eye and 20/60 in the left eye. Slit lamp exam revealed bilateral scleral injection and pigmentation present on the corneal endothelium as well as 2+ and 1+ cells by SUN classification [7] present within the right and left anterior chambers, respectively. Punctate epithelial erosions were present on both eyes and the right eye had a small amount of superficial neovascularization present on the superior cornea. Dilated fundus biomicroscopy showed mild disc edema of the left eye without signs of vitritis or posterior inflammation and normal vasculature. Posterior examination of the right eye was within normal limits. Bilateral intraocular pressure, pupil examination, and ocular motility were also within normal limits.

**Figure 1:**
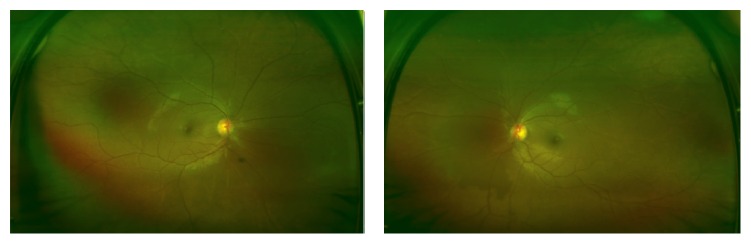
Ultra-wide-field fundus photographs of a patient with microscopic polyangiitis showing relatively normal appearing retinas in both eyes.

**Figure 2:**
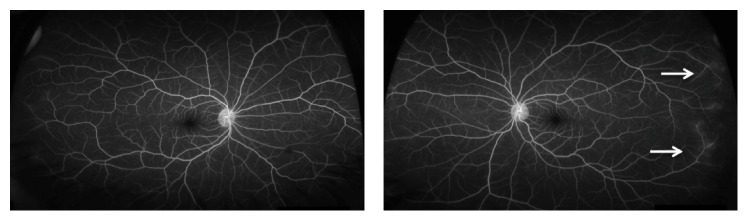
Ultra-wide-field fluorescein angiography of a patient with microscopic polyangiitis showing mild peripheral late leakage in the left eye (arrows) outside the range of standard field imaging.

The patient was diagnosed with bilateral anterior uveitis and initially treated with topical prednisolone and homatropine. One week after initiating therapy, anterior inflammation had improved and vision was 20/70 bilaterally. A broad work-up was coordinated between rheumatology and uveitis specialists. Positive results included antinuclear antibodies (ANA) (1 : 80) and p-ANCA with high myeloperoxidase activity (2.0+). The remaining diagnostic work-up—including a normal chest X-ray—was unrevealing and a diagnosis of probable microscopic polyangiitis was made. A standard field fluorescein angiography at that time revealed no signs of vasculitis. After the infectious work-up was negative, the patient was initially started on oral mycophenolate mofetil 500 mg twice daily and oral prednisone 20 mg daily, which lead to resolution of anterior inflammation and visual acuity improved to 20/20 and 20/25 in the right and left eyes, respectively. Four months later the patient had developed proteinuria (>300 mg/dL) and hemoglobinuria (moderate blood) after tapering the prednisone to 5 mg daily; thus mycophenolate mofetil was increased to 1500 mg twice daily and prednisone was increased to 20 mg daily. One year after initial presentation, ultra-wide-field fundus imaging and fluorescein angiography were first performed (when it became available at our hospital) using an Optos 200Tx (Optos PLC, Dunfermline, United Kingdom), which demonstrated mild arterial attenuation bilaterally (Figure 1) and mild temporal vasculitis of the left eye (Figure 2). The patient was restarted on a low dose of oral prednisone at that time. A repeat ultra-wide-field fluorescein angiography 3 months later revealed continued mild temporal leakage of the left eye. Due to the vasculitis and continued renal disease, rituximab therapy was initiated. One month later, the patient had no signs of inflammation or vasculitis on physical examination. The patient's care is still ongoing and we continue to follow her up closely.

## 3. Discussion

Ultra-wide-field fluorescein angiography is a new technology allowing for more extensive imaging of the peripheral retina than previous modalities have allowed. This increased field of view allows for visualization of nonperfusion and vascular leakage indicative of inflammation within more anterior areas of the retina. Wide-field angiography has been utilized in a range of retinal vascular pathologies including diabetic retinopathy, vein occlusions, sickle cell retinopathy, and uveitis [8–11].

A recent study prospectively evaluated whether ultra-wide-field imaging changes management decisions in patients with noninfectious posterior uveitis [4]. A total of 23 patients were evaluated and 4 investigators determined disease activity and management based on clinical examination, examination plus simulated 60-degree FA, examination plus ultra-wide-field color images, or examination plus wide-field FA. The addition of ultra-wide-field color images altered management in 24% of visit while the addition of the wide-field FA altered management in 51% of patients. Similar to this prior report, our patient with microscopic polyangiitis had her clinical management altered by wide-field fluorescein angiography.

ANCA-associated vasculitides include microscopic polyangiitis (MPA), granulomatosis with polyangiitis (GPA), Churg-Straus syndrome, and renal-limited vasculitis, all of which have similar features on renal histology [12]. Several classification systems exist to define and differentiate these small vessel vasculitides, although each algorithm has limitations. Our patient meets the criteria for microscopic polyangiitis based on the European Medicines Agency algorithm as she presented with glomerulonephritis and positive myeloperoxidase without surrogate markers for granulomatosis with polyangiitis [13]. There is, however, significant overlap between MPA and GPA, and organ biopsy—which we do not have for our patient—showing granulomatous changes serves as the defining pathologic difference between the diseases. Clinically, our patient lacked upper respiratory signs and had a normal chest imaging, making GPA less likely. Furthermore, MPA is primarily associated with myeloperoxidase-ANCA and GPA is primary associated with PR3-ANCA (for which our patient was negative) although approximately 20 percent of patients with MPA or GPA have the alternative ANCA [14, 15].

Microscopic polyangiitis is a rare small vessel vasculitis that can affect multiple organ systems. In a review of 85 patients meeting strict criteria for MPA (81 patient had biopsy proven diagnosis), the most common manifestations were renal disease (79%), weight loss (73%), skin involvement (62%), fever (55%), mononeuritis multiplex (58%), and arthralgias (51%) [16]. Only 1 of the 85 patients in this study was found to have ocular involvement, although it was unclear how many of these patients were evaluated by an ophthalmologist. A 2007 publication reviewed all case reports of biopsy proven MPA and found only 8 cases of MPA with ocular manifestations, which ranged from central scotomas to conjunctivitis to optic disc edema to hypopyon [17].

Given the paucity of reports of ocular involvement with microscopic polyangiitis, it is possible that ocular signs are missed in some patients with MPA. Just as our patient's vasculitis was mild and peripherally located, it is possible that some patients may have had mild signs missed by traditional imaging. Ultra-wide-field FA increases the view of the fundus, which may be able to identify more pathology and serve as a clinically meaningful tool for identifying peripheral vasculitis.

Improved visualization of retinal pathology has important clinical implications. Ultra-wide-field FA not only is useful in identifying peripheral disease, but also can be used in patients with known posterior vasculitis to determine the extent of ischemia or inflammation. By allowing simultaneous imaging of the posterior pole and periphery rather than a montage of images from different temporal and spatial locations, wide-field FA may provide a more anatomically correct image. Most importantly, enhanced peripheral vascular imaging may allow for increased sensitivity to detect pathology that could be missed on traditional FA, which can alter diagnosis, treatment, and follow-up intervals.

While the advantages of wide-field FA can improve clinical care, the limitations of the new technology should be recognized as well. Due to the wide angle of the photo and the broad depth of focus, image artifact—from lashes, cornea, or vitreous opacities—is quite common. Additionally, the central macula magnification is diminished compared to traditional FA imaging, although software can often be used to zoom in on central portions, which can partially overcome this limitation. Finally, displaying images of a spherical structure as a two-dimensional image, outer portions of the retina are stretched and distorted; however three-dimensional visualization tools are available on the machine to counteract this.

In addition to limitations of the technology itself, the interpretation of ultra-wide-field fluorescein angiography can be challenging. There is a high prevalence of peripheral vascular anatomic variation in normal eyes, which could be misinterpreted as pathologic. One recent study of eyes without peripheral disease reported a high prevalence of absent capillary detail, terminal networks, and microaneurysms with ultra-wide-field FA [18]. What could be viewed by one ophthalmologist as a normal variant might be read by another as pathologic, leading to differing opinions of treatment. Furthermore, subtle peripheral abnormalities—while truly pathological—may be so minimal as to not warrant a change in patient management. A recent study found that while peripheral vascular leakage was found in 57% of patients with clinically active uveitis, 24% of patients with well-controlled disease also showed some degree of peripheral leakage [6]. The authors point out that clinicians will tolerate a “degree of vascular leakage” in a “significant minority of patients” [6]. In the case of our patient, the peripheral leakage was mild. Immunosuppression was increased in our patient based on the entire clinical picture with the goal of treating both the peripheral retinal vasculitis and the patient's renal disease. Had the patient only had mild peripheral leakage without other signs of systemic or ocular disease activity, therapy may not have been altered.

In summary, ultra-wide-field angiography has proven to be clinically useful in a wide range of retinal diseases. The enhanced detection of peripheral pathology in wide-field FA can change patient management compared to traditional imaging—as demonstrated by our patient with microscopic polyangiitis. The role of wide-field FA, particularly in uveitis, will continue to be defined as more research elucidates the value of this new technology.
